# Speech therapy to overcome dyslexia in primary schoolers

**DOI:** 10.1038/s41598-023-31631-7

**Published:** 2023-03-22

**Authors:** Zhanar Nurseitova, Aisulu Shayakhmetova

**Affiliations:** 1 Department of Special Pedagogy, Abai Kazakh National Pedagogical University, Almaty, Kazakhstan; 2 Department of Social and Pedagogy, Sh. Ualikhanov Kokshetau University, Kokshetau, Kazakhstan

**Keywords:** Psychology, Human behaviour

## Abstract

This research was aimed to investigate changes in the reading technique and in terms of its semantic charge in primary schoolers diagnosed with dyslexia, which occur as a result of the integrated use of speech therapy techniques. The study was performed between 2016 and 2019 in 6 schools of Moscow and Almaty. It enrolled 194 and 200 children, respectively, who were examined with form I to III inclusive. The study revealed that 13% of children had reading speed disorders; they were constituted group 1. Another 11% had reading comprehension disorders; they constituted group 2. In group 1, by form III, the number of reading repetitions increased twofold. In group 2, the number of children, who read in words and phrases, increased by half; in group 1, it doubled. This research showed clear progress in children with technical dyslexia vs. those with semantic dyslexia. Based on the results, it is possible to develop a methodology for speech therapy techniques that can be suitable not only for speech therapists, but also for primary school teachers, as well as for parents of dyslectic children.

## Introduction

Today's ideology boils down to the perception of dyslexia as a reading disorder, however, having normal intellectual development^1–3^. Despite the established set of views, at present the researchers continue looking for new ways to assess the intellectual development in dyslectic subjects, although with a greater emphasis on the structural component of dyslexia. This includes both verbal and non-verbal parts, as well as the genesis of personality associated with individual and typological characteristics^4^. Dyslectic primary schoolers differ from normal peers, above all, in terms of the level of verbal intelligence. The data characterizing this direction are specifically based on the significance of verbal components associated with the cognitive mental function^5^. According to a number of authors, the greatest pedagogical results are possible in the process of mastering reading in primary school^5–7^. The reading process, along with other external and internal factors, can affect the formation of children cognitive and intellectual abilities^8^. Of particular interest are data related to the research of intellectual activity in secondary schoolers, who had different levels of reading skills. One of the most pressing issues is a clarification of the causes associated with the occurrence, dynamics and mechanisms of dyslexia^9^. In addition, the studies related to identification of the most suitable conditions and methods for successful therapy, as well as speech therapy and pediatric techniques related to the correction and prevention of dyslexia, are of certain relevance^10^. Such interest is due to statistical data. In particular, it is known that, over the past two decades, the number of children diagnosed with dyslexia has increased significantly, albeit unevenly in different regions, ranging from 3 to 25%^9,11^. For children with true dyslexia, current methods of medicine and psychology are unable to eliminate this disorder. Moreover, dyslexia is a rather serious factor that hinders the effective assimilation of educational material by students^12^. According to some experts, dyslexia may not necessarily manifest itself only for the native language^4,6–8^. The process of reading is characterized by the complication of existing discrepancies, which are expressed in the proportion of language phonemes and graphemes. Phonetic systems based on opaque spelling are characterized by a different manifestation of one phoneme through several graphemes, or vice versa, one grapheme can be associated with different phonemes^13^. In languages such as Russian, English, German and French, dyslexia is much more common^6^. In the case when an in-depth study of foreign languages is performed in regular schools, there are specific cases of dyslexia called mixed dyslexia^14^. Mixed dyslexia is associated with a set of symptoms. Firstly, this is an insufficient development of speech itself and its components, and secondly, it is a low level of perception through vision, as well as a low level of development of judgment associated with visual and motor function. In specialized schools, students study not one, but two foreign languages at once. In most cases, dyslectic children come to such schools accidentally, not by their own choice^15^. As a rule, this is due to the lack of passing tests when entering the school. So, dyslexia is quite common among children; it is a persistent disorder that negatively affects schoolers’ academic performance. In this regard, it is necessary to develop new medical, psychological, speech therapy techniques for teaching dyslectic children. Involving parents in teaching children with dyslexia does not give quick and reliable results^16^. Another and better results can be achieved if various specialists are involved, for example, speech therapists, as well as teachers of native and foreign languages. The educational process at school is based on the concept that children learn to read quickly and understand the essence of the text they read. Therefore, the quality of the student's understanding of assignments, as well as various control and independent work, depends on how well the reading skill is developed. For school age, reading is of great importance, since it helps children to learn about the world around them, including that in their free time. Recently, social networks and mobile applications related to the global digitalization of society have played an increasingly important role in this^11^. Therefore, if dyslexia presents and progresses, the child should be expected to have not only poor academic performance, but also maladaptation in society.

Global practice in the research of dyslexia is associated with two areas. In Western countries, dyslexia is dominantly considered as a disorder resulting from improper mastery of reading technique^8,15^. At the same time, the main parameters of reading technique are reading speed and correctness. The disadvantage of this approach is one-sided perception of the reading technique, without taking into account the conceptual component of this process. According to this opinion, the difficulties that arise in understanding the meaning when reading are primarily related to disorders in accessing the information in the memory. Therefore, the root cause lies in the child's cognitive perception. The second approach is mainly related to the research on the territory of the former Soviet Union; it is aimed at the very perception of the word, its form and meaning. Other studies specify an isolated type of reading disorders and its semantic component^2^. At the same time, there are no detailed studies on this subject. This formed the basis for the present study. In addition, the authors proposed speech therapy techniques that can significantly improve reading performance in primary schoolers.

This research was aimed to investigate changes in reading technique and in terms of its semantic charge in primary schoolers diagnosed with dyslexia. Study objectives were as follows: (a) to track the parameters of the technical reading component over time; (b) the same for the semantic reading component; (c) to test the speech therapy techniques to improve reading performance in dyslectic children. The authors believe that the introduction of the proposed speech therapy techniques will significantly improve reading performance in children. A limitation of this study may be the application of these techniques to Russian-speaking and Kazakh-speaking children.

## Materials and methods

### Population

This longitudinal study involved 194 and 200 primary schoolers attending 3 regular schools in Moscow (Russia) and Almaty (Kazakhstan, also 3 schools), respectively. The Russian population included 100 girls and 94 boys; the Kazakh one—100 boys and 100 girls. The classes were held in Russian and Kazakh. The study did not take into account gender differences. The mean age of children was 6.1 ± 1.1 years. The participants were allocated to groups after the initial examination; it is detailed in the "Results" section.

### Study design

This study was complex: in addition to investigating the particularities of dyslexia in primary schoolers, we explored the results of the use of speech therapy techniques on children’s reading skill. The study enrolled children, whose parents gave written consent (signed a contract). The contract revealed the essence of the study, described in detail the possible consequences; it was proposed to try a promising new method of speech therapy for children. The study enrolled all children, whose parents had signed the contract. At baseline, dyslectic children were not identified specifically; all children, who later entered the dyslectic group, were revealed after initial testing. This formed the basis for further allocation to groups. The study consisted of two parts. The first part provided the analysis results of dyslexia over time when children studied in forms I–III of a regular school; the second part discussed the results of dyslexia course, taking into account the use of speech therapy techniques. For the Kazakh population, classes were held in Kazakh and Russian, as many children knew Russian quite well.

### Ethical aspects

The study was performed in accordance with the international standards of ethics and morality. Anonymity and confidentiality of the information related to participants was observed.

### Methods

The research was conducted at the same time, from 11 o'clock in the morning, when the children were not yet tired, but a considerable time had already passed after sleep. Thus, we tried to reduce the possible inhibitory effect of somnolence or fatigue on the reading process. During primary testing, children read special texts, the moments of beginning and the end of reading was recorded, due to which the accuracy needed for measurements was achieved. The definition of reading speed included the time period, in which the child read a certain text. Different reading parameters were taken into account, such as reading method, repetitions and mistakes. After testing, the children retold the text, and simultaneously such parameters as unfragmentation of the text (its integrity and connection between different parts) were evaluated. Analysis was based on results obtained for study participants vs. those in normal peers; it considered reading speed and understanding the meaning of what was read.

### Speech therapy approaches

This experiment had the following hypothesis: in the case of a positive outcome, the results can be used in educational activities. Many teachers note that, due to the low awareness of dyslexia, they tend to think about children with this diagnosis that such schoolers are lazy, low-motivated, or even have insufficient mental abilities. The experiment was based on the following speech therapy techniques and pedagogical practices:

1. Emphasis on the correct pronunciation, the accuracy of sound articulation. At the same time, visual imagery and tasks related to verbal accompaniment of actions constituted its basis. The pronunciation of sounds in native languages (Russian in Russia, Kazakh in Kazakhstan) was compared, while monitoring the position of the organs responsible for articulation. To do this, the mirror was used to control position of the lips, as well as the tongue. In addition, tactile as well as kinesthetic sensations were taken into account. The studied sounds were accompanied by the movement of the hand, which seemed to reproduce it. It was possible to work with problematic (hissing, whistling) sounds using special exercises of articulatory gymnastics, with parallel observation in the mirror.
2. The exercises related to the development of speech breathing implied short nasal inhalation and long exhalation through the mouth. During expiration, short phrases were pronounced and accompanied with movements.
3. Work with different types of motor skills. This includes general, manual and articulatory motor skills. Coordination type was hand-eye. Before each lesson, training was performed in the form of logorhythmic elements: children recited short poems accompanied by movements of the arms and corpus. Such gymnastics involves muscles of the lips, tongue, neck and upper shoulder girdle. In addition, students were offered additional tasks, such as to draw the second half of either a letter or an object, from top to bottom, then from left to right. They also drew labyrinth paths to develop spatial coordination.
4. Exercises were also used to develop visual analysis and spatial coordination. These exercises were included to the study because children diagnosed with optical dyslexia could have difficulties recognising letters under complicated conditions. Children determined the correct letter from several options: in the usual and mirror position, in an inverted position. In addition, other variations were used, for example, for similarly written Cyrillic letters: either И and H, or Б and B. In the version of the Latin alphabet used in Kazakh, these were O-Q, v-w, o-c, R-P, r-n-m and others. Similarly, Kazakh children were offered tests in Kazakh. In addition, the participants were offered to find letters consisting of the same number of components, search for words in single stream of letters, which was differently oriented in space. Children wrote graphic dictations.
5. Exercises aimed at developing visual attention. Children read isographs, also they read certain-type syllables (for example, consisting of capital letters, vowels and consonants, or highlighted in a certain font color) in the proposed table from left to right. Corrective cross-sections were performed, the children tried to find dual images in the proposed pictures. Also red–black tables and Schulte tables were used. At the same time, different manifestations of attention were taken into account, such as concentration of attention, its distribution and switching from one object to another.
6. Special techniques were used that aimed at developing the perception of sounds, as well as their analysis, synthesis, and presentation. It was taken into account that the highest mental function can be brought up by going through the following stages: the materialization of any action (with obligatory visualization), the performance of these actions inside, the presentation of actions received from the outside and their implementation up to automatism.
7. Visualization was performed according to Davis method. The first part included an assessment of the ability to perceive the environment. To do this, the child was asked to see the surrounding world by his/her mental eye, followed by the second part, in which the child learned to consciously turn on or off his/her spatial orientation. During the exercise, the child was asked to find a personal point of adjustment of his/her spatial orientation. The final stage of the method was the transition of thinking from symbols, which is typical for dyslectic children, to thinking associated with words. This provided a transition from visual to verbal thinking, which could make it easier for children to read.

Finally, we have corrected mistakes caused by dyslexia when pronouncing words or sentences. For this, the following speech therapy techniques were adapted:

- acrophonic reading, in which a new word was composed from the first letters of another word, or a picture corresponding to it was selected;
- reading the words by numbers. To do this, the letters of the main word were numbered, and children were asked to compose a new word using them;
- reading the words based on the sequence of figures corresponding to them;
- in the proposed group of words, the children were asked to find the extra one, initially at the intermediate level, then at difficult levels, for example, in a square that was filled with letters;
- making up a sentence or a text from the letters of the deformed sentence. In order to develop anticipation in children, they read with a lattice or using a window that had a slot on the left side (to prevent anticipation);
- reading the words or their combinations based on letters partially covered from above or below;
- reading inverted words and whole phrases;
- exercises aimed at reducing the low rate of children's activity: ‘Flash’, ‘Tug’.

A characteristic feature of the speech therapy techniques and methods used was their complexity, that is, the maximum possible number of analyzers (vision, hearing, tactility, kinesthetics) were included in the learning process. In addition to the standard handouts and exercise games, information technology was used in the form of passing tests on tablets and smartphones. Dyslectic children are characterized by the rapid onset of fatigue, easy distraction. In this regard, there is a need in frequent change of the perspective of attention to different forms of activity and work, taking into account the individual performance of the child, as well as his/her speech and motor skills. Another feature is the portioning of information, the need for a gradual complication of tasks and the supplied speech material.

### Statistical analysis

Statistical analysis was performed using Microsoft Excel 2016. For each of the studied parameters, its frequency among children was calculated as a percentage as the schoolers progressed from form I to form III inclusive. Significance of differences between parameters was determined using Student's t-test. The differences were significant at P ≤ 0.05. The tables show mean values between the groups of children from Russia and Kazakhstan, since no significant differences were found between two populations.

### Ethics approval

The authors declare that the work is written with due consideration of ethical standards. The study was conducted in accordance with the ethical principles approved by the Ethics Committee of Abai Kazakh National Pedagogical University and Sh. Uvalikhanov Kokshetau University (Protocol No. 76 of 13.09.2022).

### Consent to participate

Informed consent was signed by participants.

## Results

Retrospective analysis able to give concrete results at the end of form III was based on processing of the raw data. During this period, dyslectic children can be distinguished from the whole study population. The number of such children was significant: 27% in Russian population (52 children) and 24% in Kazakh one (48 children). The results obtained have no statistical differences (P ≥ 0.05). This suggests that the frequency of dyslexia was approximately equal in both populations. In the Russian population, 13% of children had isolated reading speed disorders; they constituted study group 1 (25 children). Another 11% had reading comprehension disorders; they formed study group 2 (21 children). Similarly, in the Kazakh population, isolated reading speed disorders and reading comprehension disorders were found in 12% (24 children) and 10% of children, respectively (20 children). At the same time, there were no statistically significant differences for tests in Russian and Kazakh in the Kazakh population. There were no differences in the frequency of dyslexia types in both populations (P ≥ 0.05). The total number of children from both countries in group 1 was 49, in group 2 it was 41. A small number of children remaining in each population had combined reading speed and comprehension disorders and were not included in this study.

At the same time, there were differences in reading speed for study group 1, and in reading comprehension in study group 2. Both groups included children with mild, moderate, and severe dyslexia. Mild dyslexia was defined as Mi ≥ m + σ, moderate as Mi ≥ m + 1.3σ, and severe as Mi ≥ m + 2.0σ.

In both study groups of both populations, the parameters of reading technique were compared. Their values are given in Table 1, without results for form II, because they are intermediate.

**Table 1:** Parameters of reading technique in forms I–III (Russian and Kazakh population, mean), % + (the number of children).

Group number	1		2		3		4		5	
	I	III	I	III	I	III	I	III	I	III
One (N = 49)	27.9 (13)	44.9 (22)	38.9 (19)	**86.0 (42)**	**48.9 (24)**	7.9 (4)	5.4 (3)	4.5 (2)	5.4 (3)	**12.3 (6)**
Two (N = 41)	55.8 (23)	79.6 (33)	**74.0 (30)**	94.45 (39)	19.4 (8)	3.1 (1)	3.1 (1)	3.1 (1)	3.1 (1)	3.1 (1)

Significant changes are highlighted in bold (P ≤ 0.05), I, III—forms; 1—number of words per minute; 2—ability to read words or combinations of words; 3—ability to read with syllables; 4—reading mistakes; 5—reading repetitions.

The results compared have releaved differences between two groups that started from form I. In group 1, the number of children reading in syllables by form III decreased significantly, sixfold (P ≤ 0.05 between forms I and III); in group 2 it was also sixfold (P ≤ 0.05), however, the baseline number of such children was lower in this group. In group 1, by form III, the number of reading repetitions increased twofold (P ≤ 0.05), while group 2 had no statistically significant changes (P ≥ 0.05). On the other hand, in group 2, the number of children, who read in words and phrases, increased by half (P ≤ 0.05). In group 1, this number doubled (P ≤ 0.05).

In study group 1, the majority of children correctly read words using the syllabic method, while children in group 2 (74% of them) mainly used more advanced methods, such as reading in words and their combinations. In some cases, children in group 2 read even faster than mean reading speed for respective age norm. Starting from form I, children in group 1 demonstrated a greater number of mistakes and repetitions vs. children in group 2 (Table 1, P ≤ 0.05). At the end of form III, the majority of children in both groups managed to master the technique of fluent reading in words and their combinations. There was no relationship between the text complexity and the number of mistakes (P ≥ 0.05). In group 1, the number of repetitions increased in proportion to complexity of the text message. Possible reasons for this were the problems that children had while working with verbal visual symbols. Analysis of the children’s reading repetitions allowed to classify them based on the frequency. In most cases, repetitions occurred at the beginning of a word (‘вв-вынec’ and ‘пп-пpивeл’, transcribed as [vv-vynes] and [pp-privel], and translated as ‘cc-carried out’ and ‘ll-led’, respectively). Children had problems with the integrity in perception of the words, which were new for them. The next group in terms of frequency consisted of repetitions that occurred when the initial syllable was incorrectly interpreted, for example, ‘пpи-пpoбил’ and ‘пpo-пpинec’ ([pri-probil] and [pro-prines], translated as ‘hammered-punctured’ and ‘carried-brought’, respectively). The third group consisted of repetitions that occurred when the word was unfamiliar and children inserted a familiar one instead, for example, ‘внyк-внyк пocтpoил’ ([vnuk-vnuk postroil], translated as ‘the grandson has built’). The rarest group of repetitions consisted of those that occurred when children moved to the next line, but continued thinking about the previous one.

All these repetitions were associated with disorders that result from misperception of words, as well as difficulties with visualization. This was verified by analyzing the data obtained from the study of visualization. If the child read slowly, there were problems with three-letter visualization symbols to find them among other symbols. The schoolers had problems in identifying the pattern and finding this pattern. This resulted from their visual problems. Therefore, possible changes in children with dyslexia of different grades were analyzed and compared to the normal values. For those with severe dyslexia, deterioration was recorded during all 3 school years; in moderately dyslectic schoolers, this manifested itself in form II, while mild dyslexia became evident only in form III. From this we can conclude that the degree of dyslexia is directly related to the time of its manifestation. The schoolers, who had difficulties in understanding the text, usually coped with reading words and their combinations within the normal range, but when reading short words they often made mistakes if these words had a visual resemblance to each other (‘нo-oн’, ‘cтaлo-cтaдo’ [‘no-on’, ‘stalo-stado’ translated as ‘but-he’, ‘became-herd’, respectively). Finally, a comparative analysis was conducted; it showed how the children understood the message they read using retelling analysis (Table 2).

**Table 2:** Retelling parameters, scores, mean for Russian and Kazakh populations, % + (the number of children).

Group title	Quality of retelling		Adequacity of the conveyed meaning		Probability of programming		Formatting in terms of vocabulary		Formatting in terms of grammar	
	Form I	Form III	Form I	Form III	Form I	Form III	Form I	Form III	Form I	Form III
Study group 1 (N = 49)	12.3 (6)	**23.0 (11)**	7.9 (4)	10.7 (5)	7.9 (4)	10.7 (5)	10.7 (5)	10.7 (5)	7.9 (4)	7.9 (4)
Study group 2 (N = 41)	12.6 (5)	**10.7 (4)**	3.1 (1)	**0.0 (0)**	3.1 (1)	5.0 (2)	5.0 (2)	5.0 (2)	5.0 (2)	5.0 (2)

Significant values are in bold.

The data obtained show that children in group 2 at the beginning of form I had low scores for text retelling, and this persisted until form III, when these parameters became even lower (in group 2, the quality of retelling decreased 1.1-fold, P ≤ 0.05; adequacy decreased twofold, P ≤ 0.05). Group 1 demonstrated improvements: the quality of retelling improved twofold by form III (P ≤ 0.05). This is due to the fact that in the form II the texts became larger and more complex. The majority (70%) of children in group 2 were unable to retell the text read, while for group 1 the proportion of such children was 3 times lower (23%). However, by the end of form III, only group 2 included children who could not retell the text. The following common points remained: retelling of the text by fragments, low number of connected storylines, and interpretation of events that differs from that in the story. This indicates a low ability to build a complrehensive and coherent retelling in group 2. By the end of form III, the children in group 1 understood the texts; the schoolers made progress along with appropriate complication of texts. Despite the fact that some details or words were skipped, it did not affect the accuracy of the retelling itself. There were also no mistakes in vocabulary and grammar. Children in group 1 were characterized by understanding of the texts they read. This progress was achieved with active using a set of speech therapy techniques, which made it possible to work taking into account the individual characteristics of each child. However, despite this, the results depended on the type of dyslexia; in group 2, they were significantly lower. Based on the data obtained, it can be concluded that dyslexia may be of two types—technical and semantic.

## Discussion

The majority of children (61%) in group 1 were at the stage of reading in syllables, while children in group 2, on the contrary, read mainly in words and their combinations, while only a quarter of them (26%) read with syllables. In group 1, only some children (39%) read in words and phrases, while, in group 2, 74% of the children read in words. Children in group 1 had lower level of text retelling compared to group 2; this was seen starting from form I (70% versus 23%). In form III, this pattern was preserved. Some children in group 1 already in form I demonstrated agrammatisms, which aggravated the situation in form III, as the text structure became more complicated, and mistakes caused by incorrect perception of visual symbols persisted. First of all, children in group 1 demonstrated decrease in the parameters of visual memory, as well as auditory and verbal memory during the progression from form I to form III. Children in the Kazakh population did not show significant differences in understanding and reading speed between Russian and Kazakh languages. Perhaps this was due to the mixed languages of communication in their families.

The main goal of a speech therapist is to correct mistakes, as well as to prevent them. Mistakes include those associated with visual disturbances in perception, ideas of time and space^17^. Correction is achieved by including various kinds of activities in the work with the child, or game learning, as well as tasks and exercises. The latter case uses an interactive whiteboard.

First of all, the work with a child should take into account that reading today is the basis, which forms the foundation of knowledge about the world around^18^. Reading gives the opportunity to learn and form any actions of a universal type in the entire learning process^19^. Dyslexia is most often defined as a reading disorder, which is associated with unformed mental functions that provide it^20^. In such case, a person is unable to recognize the words correctly, so he or she encounters difficulties in mastering the basics of spelling. Further, secondary signs join this, such as misunderstanding of the text or lack of reading experience, which is mainly due to the underdeveloped phonology of the language^21^. At the same time, according to a number of data, cognitive abilities remain intact, but difficulties in cognition are present even under full-fledged conditions of the educational process, including that in primary schools^22^. On average, global number of children with normal intelligence, but diagnosed with dyslexia, is about 5%^23^. However, if a child has diagnosis of mental retardation and speech disorder, then the frequency of dyslexia increases by 2–5 times, up to 50%^24^.

If a child already has dyslexia when he or she enters school, this disorder does not develop in an isolated way. Dyslexia also may have other causal factors, such as complicated pregnancy or genetic predisposition. However, such causes can be diagnosed even in early childhood^25^. At the same time, a large number of social factors can also influence the child’s mental processes, leading to increased anxiety, social maladjustment, or it can be indifference of parents to the child’s development, for example, to the development of his/her reading technique, or incorrect pedagogical approach to preschool learning^26^. Another reason may be bilingualism in the family, different nationalities of the parents. Different authors distinguish a different number of dyslexia types, but six are most often mentioned^27–29^. These include phonemic, semantic, agrammatic, mnestic, optical and tactile types of dyslexia. The mechanisms that cause dyslexia are related to further problems with speaking and writing. Elimination of dyslexia requires the support of a speech therapist^30^. Moreover, primary school teachers should have speech therapy knowledge so that they become able to help to at least single out dyslectic schoolers from the general population. The research being described showed a clear progress in children with technical dyslexia vs. children with semantic dyslexia, which confirmed data from other regions. At the same time, such studies enrolled Russian-speaking and Kazakh-speaking children together for the first time, and the results obtained showed the unreliability of differences in the number of children with different forms of dyslexia. In addition, no differences were found in the reading comprehension and speed between Kazakh children, who were offered tests in Kazakh. On the other hand, for the first time a set of speech therapy techniques gave positive results that indicate the undoubted benefits of this approach. This approach is necessary for all primary schoolers and applies not only to children with special educational needs, but also to those who can normally be considered healthy. Another important point is that preschool learning of reading largely determines the child's mastery of the reading skill. According to our data and the colleagues’ results, the greatest problems with reading arise in children, who have low reading experience before school or low motivation for reading. In this regard, based on the data obtained, we recommend the age of 5 years to start reading classes. These activities are important as children learn to adjust and control their reading process. It is known that if a child of preschool age has difficulties in remembering letters, then he or she is more likely to have a predisposition to dyslexia^31^. In order for someone to learn reading correctly, the teacher needs to take into account the child’s level of reading before entering the school. In accordance with this, it is necessary to select the material that will develop reading in an individual child. If parents knew about disorders in the formation of reading skills, they have the opportunity at the right time to seek advice and correction from a speech therapist. The results obtained can be used to develop guidelines for teachers, speech therapists, in order to correct reading skills in children. However, these results may not have a significant positive effect on children with moderate and severe dyslexia, as shown in the study being described. Moreover, children with semantic dyslexia demonstrate low results, even taking into account an integrated approach. Future research may focus on finding methods to detect dyslexia in younger children, such as in preschool age.

## Conclusion

A set of speech therapy techniques made it possible to improve the dyslexia course over time, up to improvement in children with mild, and, partially, moderate disorder. Those with severe dyslexia demonstrated improvements, but the changes were insufficient. A change in the approach to educational and educational process turned out to be of great importance; this yielded results in conjunction with a set of speech therapy techniques. This made it possible to implement methods for correcting dyslexia, improved the children’s motivation to master the language basics. At the same time, there were no differences between the Russian and Kazakh populations. This indicates the commonality of dyslexia development, apparently, regardless of language affiliation. The practical value of the data obtained lies in the fact that based on the results it is possible to develop a methodology for speech therapy, which can be suitable not only for speech therapists, but also for primary school teachers. In addition, if the manuals are written in a plain language, then the parents of dyslectic children can use them. However, it should be borne in mind that work will be effective primarily with children with mild dyslexia. On the other hand, early identification of dyslectic children is of paramount importance. This can increase the effectiveness in correcting this disorder. The main number of mistakes made by children during testing was related to distortions of initial syllables, repetitions of initial syllables or their replacement of subsequent syllables. In addition, the children with problems in identifying visual verbal symbols showed a very low reading speed, although the meaning was preserved. The results obtained lead to the need for regular examination of children by speech therapists, starting from the older groups in kindergartens, which should be systematic, comprehensive and accessible. It is necessary to focus further on identifying children with severe dyslexia. It is also crucial to develop a set of methods to alleviate the symptoms of dyslexia and improve motivation and reading parameters in these children. Successful work with children with mild (or, to a lesser extent, moderate) dyslexia requires a set of measures. These include:

A. Passing dyslexia tests. Parents can carry out such tests at home via the Internet. A speech therapist can subsequently correct the results obtained by parents.
B. Creation of methodological manuals based on the results obtained in this study. This is one of the further research directions.
C. Introducing these tests in school and working with children with mild and moderate dyslexia.

## Data Availability

Data will be available on request by the corresponding author (Z. Nurseitova).
